# The Role of the ATP-Binding Cassette A1 (ABCA1) in Human Disease

**DOI:** 10.3390/ijms22041593

**Published:** 2021-02-05

**Authors:** Leonor Jacobo-Albavera, Mayra Domínguez-Pérez, Diana Jhoseline Medina-Leyte, Antonia González-Garrido, Teresa Villarreal-Molina

**Affiliations:** 1 Laboratorio de Genómica de Enfermedades Cardiovasculares, Dirección de Investigación, Instituto Nacional de Medicina Genómica (INMEGEN), Mexico City CP14610, Mexico; ljacobo@inmegen.gob.mx (L.J.-A.); mdominguez@inmegen.gob.mx (M.D.-P.); dianajhos18@gmail.com (D.J.M.-L.); agonzalezg@uchicago.edu (A.G.-G.); 2 Posgrado en Ciencias Biológicas, Universidad Nacional Autónoma de México (UNAM), Coyoacán, Mexico City CP04510, Mexico

**Keywords:** ATP-binding cassette transporter A1 (ABCA1), cholesterol homeostasis, reverse cholesterol transport, HDL-C, dyslipidemia, type 2 diabetes, microparticles

## Abstract

Cholesterol homeostasis is essential in normal physiology of all cells. One of several proteins involved in cholesterol homeostasis is the ATP-binding cassette transporter A1 (ABCA1), a transmembrane protein widely expressed in many tissues. One of its main functions is the efflux of intracellular free cholesterol and phospholipids across the plasma membrane to combine with apolipoproteins, mainly apolipoprotein A-I (Apo A-I), forming nascent high-density lipoprotein-cholesterol (HDL-C) particles, the first step of reverse cholesterol transport (RCT). In addition, ABCA1 regulates cholesterol and phospholipid content in the plasma membrane affecting lipid rafts, microparticle (MP) formation and cell signaling. Thus, it is not surprising that impaired ABCA1 function and altered cholesterol homeostasis may affect many different organs and is involved in the pathophysiology of a broad array of diseases. This review describes evidence obtained from animal models, human studies and genetic variation explaining how ABCA1 is involved in dyslipidemia, coronary heart disease (CHD), type 2 diabetes (T2D), thrombosis, neurological disorders, age-related macular degeneration (AMD), glaucoma, viral infections and in cancer progression.

## 1. Introduction

Cholesterol is an essential biomolecule, involved in a wide array of physiological and pathological processes. In the plasma membrane, changes in free cholesterol content and phospholipid species modulate signaling of multiple receptors [1]. A physiological free cholesterol/phospholipid ratio in cellular membranes is necessary to maintain membrane fluidity [2], and altered membrane fluidity adversely affects the conformation and function of certain integral membrane proteins that can be inhibited by a high free cholesterol/phospholipid ratio [3]. Excess plasma membrane cholesterol also disrupts the function of certain signaling molecules that normally reside in non-raft domains. In addition, excess intracellular cholesterol levels can also cause toxicity by mechanisms including intracellular cholesterol crystallization, oxidation of cholesterol to oxysterols and triggering of apoptotic signaling pathways [4].

**Figure 1:**
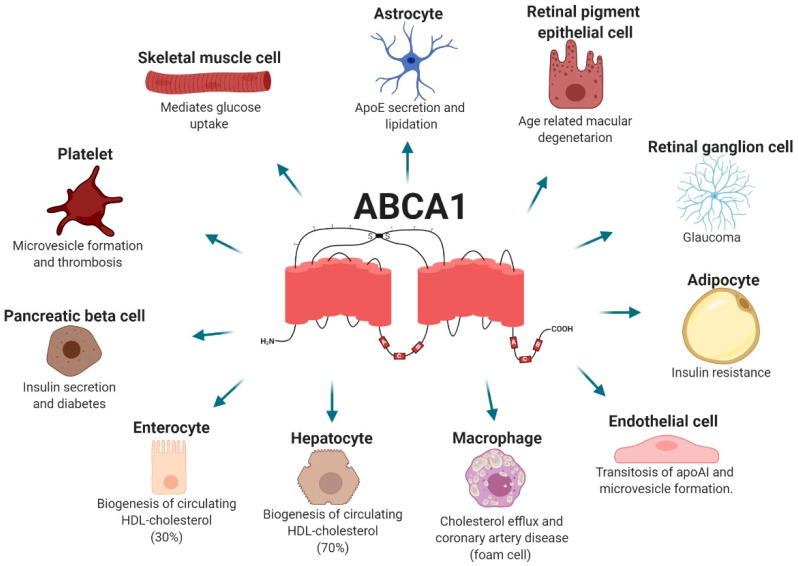
ATP-binding cassette transporter A1 (ABCA1) functions in different cell types and associated diseases. ABCA1 is widely expressed and participates in a broad array of physiological and pathological processes. ApoE: apolipoprotein E; HDL: high-density lipoproteins; apoAI: apolipoprotein I.

One of several proteins involved in cholesterol homeostasis is the ATP-binding cassette transporter A1 (ABCA1), a transmembrane protein widely expressed in many tissues where it may have many different functions. Its most studied function is the efflux of intracellular free cholesterol and phospholipids across the plasma membrane to combine with apolipoproteins, mainly apolipoprotein A-I (ApoA-I), forming nascent high-density lipoprotein particles (HDLs), the first step of reverse cholesterol transport (RCT) [5]. RCT is the process by which the body removes excess cholesterol from peripheral tissues and delivers this cholesterol to the liver, where it is redistributed to other tissues or removed from the body by the gallbladder. HDL-cholesterol (HDL-C) particles are the main lipoproteins involved in this process [6]. In addition to HDL-C formation, ABCA1 regulates cholesterol and phospholipid content in the plasma membrane and is involved in microparticle formation and thus in cell signaling. For all these reasons, it is not surprising that altered cholesterol homeostasis may affect many different organs and is involved in the pathophysiology of a broad array of diseases (Figure 1). The present review focuses on the role of the ABCA1 cholesterol transporter in human disease.

## 2. Global ABCA1 Deficiency: Tangier Disease

Tangier disease (TD) is a rare autosomal recessive disease caused by homozygous or compound heterozygous loss of function variants in both alleles of the *ABCA1* gene (OMIM #205400). TD is characterized by severe deficiency or absence of circulating HDL-C particles and accumulation of cholesteryl-esters in cells throughout the body, particularly in the reticuloendothelial system [7,8]. The major clinical signs of TD are very low HDL-C levels (<5 mg/dL), hyperplastic yellow orange tonsils and hepatosplenomegaly; while peripheral neuropathy occurs in approximately 50%, and premature coronary heart disease (CHD), occurs in 30 to 50% of TD patients [9,10,11]. Carriers of a single *ABCA1* mutation (heterozygotes) have variable reductions in plasma HDL-C levels and a variable increased risk for CHD [12]. Other less frequent symptoms include corneal opacity and hematologic manifestations, such as thrombocytopenia, altered platelet morphology and function, mild bleeding tendency, reticulocytosis, stomatocytosis and hemolytic anemia [13].

Macrophages and other cells from TD patients are overloaded with cholesterol (foam cells) because the ABCA1-mediated efflux of cellular free (unesterified) cholesterol and phospholipids to ApoA-I is defective [14]. These foam cells play a crucial role in the pathogenesis of atherosclerosis and CHD. However, it is not clear why not all TD patients develop premature CHD. A review of 185 TD cases reported that 51% of patients aged 40 to 65 years had premature CHD and suggested that reduced low-density lipoprotein-cholesterol (LDL-C) levels in TD patients provide cardiovascular protection, while TD patients with normal LDL-C levels are likely to develop premature CHD [10]. A more recent review reported angina in 24.8%, and other vascular diseases in 21.8% of TD cases. Patients with CHD had a higher mean age, and while total cholesterol and LDL-C levels were higher in CHD than in non-CHD TD patients, the differences were only statistically significant in women [15]. The presence of small-dense LDL-C particles in some TD patients are also thought contribute to the development of CHD [10,15].

## 3. ABCA1 and Plasma Lipid Levels

### 3.1. ABCA1, Reverse Cholesterol Transport and Plasma HDL-C Levels

The key role of ABCA1 in RCT and HDL metabolism is evident as *ABCA1* gene mutations causing Tangier disease are associated with extremely low plasma HDL-C levels, a characteristic feature of the disease [16,17]. Murine tissue-specific knockout (KO) models have shown that cholesterol efflux via hepatic *Abca1* is responsible for 70% [18], whereas intestinal *Abca1* is responsible for 30%, of the biogenesis of HDL-cholesterol [19], thus leaving only a minute fraction of total cholesterol efflux to arterial wall macrophages.

In the first step of RCT, ABCA1 exports excess cellular cholesterol and phosphatidylcholine (PC) to circulating lipid-free ApoA-I [20]. This generates nascent HDL, a bilayer fragment formed by 200 to 700 lipids wrapped by two to four ApoA-I molecules [21,22]. Two different models have been proposed to explain how nascent HDL-C particles are formed. According to the direct loading model, ABCA1 transfers lipids to ApoA-1 directly while it is bound to the transporter. In the indirect model, the phospholipid translocation activity of the ABCA1 protein forms specific membrane domains, and ApoA-I acquires lipids through these domains. The existence of two types of ApoA-I binding sites on the plasma membranes of cells expressing ABCA1 (a high-affinity/low-capacity binding site and a low-affinity/high-capacity binding site) supports the indirect model [23,24]. This model is also supported by the observation that ApoA-I alone can bind to high curvature liposomes and spontaneously form discoidal HDL particles in vitro [25]. Recently, in baby hamster kidney/ABCA1 cells, Ishigami et al. reported that trypsin treatment causes rapid release of PC and cholesterol, suggesting that these lipids are temporarily sequestered at trypsin-sensitive sites on the surface of cells in an ATP-dependent manner. Thus, these sites may be the large extracellular domains (ECDs) of ABCA1, and the lipids may be temporarily sequestered within these ECDs during nascent HDL formation [26]. Although further studies are required to establish the molecular details of the mechanistic links between the ECDs of ABCA1 and the known functions of the transporter, it is clear that ABCA1 function is the first and a crucial step for HDL-C formation.

### 3.2. ABCA1 Gene Variation Is Associated with HDL-C Levels

*ABCA1* is a highly polymorphic gene located on human chromosome 9 (9q31.1) containing 50 exons [27]. According to the NCBI genetic variation database (https://www.ncbi.nlm.nih.gov/SNP), over 5000 polymorphisms have been reported in or near this gene. Several of these variants (intronic, missense and located in the promoter region) have important effects on the expression and function of the ABCA1 protein [28,29].

Both rare and common genetic variations in *ABCA1* contribute to circulating levels of HDL-cholesterol in population-based studies. Genome-wide association studies (GWAS) have consistently identified *ABCA1* as a locus associated with HDL-C levels in various ethnic groups [30,31]. Three nonsynonymous *ABCA1* polymorphisms have been extensively studied in terms of their associations with plasma lipid levels and CHD risk over the past two decades: rs2230806 (R219K) [32,33,34,35,36,37], rs2066714 (I883M) [33,35,38,39,40] and rs2230808 (R1587K) [33,38]. A recent meta-analysis confirmed the association of these three variants with plasma lipid levels [27]. Notably, a functional *ABCA1* missense variant (rs9282541; R230C) that was found to be private to the Americas was strongly associated with low HDL-C levels in Mexican mestizos and Native American populations [41,42]. This variant is of particular interest because it decreases cholesterol efflux capacity of the protein, is relatively frequent in the Mexican mestizo population (minor allele frequency is approximately 10%) and the sole presence of the risk allele explains almost 4% of plasma HDL-C variation.

Few studies have reported interactions between *ABCA1* gene variants and dietary macronutrient proportions affecting plasma lipid levels. In the Mexican population, two independent studies observed that the inverse correlation between carbohydrate intake and HDL-C concentrations was of higher magnitude in premenopausal women bearing the *ABCA1*/R230C variant [43,44]. Jacobo-Albavera et al. also reported that premenopausal women carrying the *ABCA1*/R230C risk allele, and consuming lower fat and higher carbohydrate dietary proportions, showed an overall unfavorable metabolic pattern including lower HDL-C levels. This suggests that gene-diet interactions play a role in inter-individual lipid level variations and may provide information useful to design diet intervention studies. In this regard, a study in Mexican individuals with hyperlipidemia reported that those bearing the *ABCA1*/R230C variant showed lower HDL concentrations and were better responders to a dietary portfolio treatment designed to increase plasma HDL-C concentrations [45]. Altogether, these studies demonstrate the relevance of the *ABCA1*/R230C variant on the regulation of HDL-C levels in the Mexican population.

### 3.3. ABCA1, miRNAs and HDL-C Levels

MicroRNAs regulate the expression of most genes associated with HDL metabolism, including *ABCA1*, *ABCG1* and the scavenger receptor *SRB1*. This implies that miRNAs regulate HDL biogenesis, cellular cholesterol efflux and HDL-C hepatic uptake, thereby controlling all steps involved in RCT [46].

Several miRNAs targeting *ABCA1* and regulating HDL-C plasma levels have been identified. miR-33a and miR-33b are embedded in intronic regions of the *SREBF2* and *SREBF1* genes which encode the SREBP2 and SREBP1 transcription factors that control the expression of genes involved in cholesterol and fatty acid synthesis [47,48]. Both miR-33a and miR-33b are coregulated with their host genes and repress gene programs that oppose SREBP functions like cholesterol efflux and fatty acid oxidation. The physiological relevance of miR-33 targeting of *ABCA1* was initially demonstrated using miR-33 inhibitors, which caused a two-fold increase of cholesterol efflux from hepatocytes to ApoA-I in vitro [47] and a 30% increase of plasma HDL-C levels in mice [48]. Moreover, targeted deletion of miR-33 caused a 25% increase in plasma HDL-C in male, and a 40% increase in female miR-33 null mice [49]. 

*ABCA1* has a long 3′ UTR (>3.3 kb), making it especially susceptible to miRNA post-transcriptional control. miR-758 [50], miR-26 [51] and miR-106b [52] have also been found to repress ABCA1 and cholesterol efflux in vitro. In addition, two independent research groups stated that miR-144, an intergenic miRNA present in the miR-451 bicistronic cluster, also targets liver ABCA1 and modulates HDL-cholesterol plasma levels [53,54]. Moreover, in vivo activation of the farnesoid X nuclear receptor (FXR) increased hepatic miR-144 levels, which, in turn, decreased hepatic ABCA1 and plasma HDL-C levels. In vitro miR-144 overexpression decreased both cellular ABCA1 protein and cholesterol efflux to lipid-poor ApoA-I, while in vivo overexpression reduced hepatic ABCA1 protein and plasma HDL-cholesterol. Conversely, hepatic ABCA1 protein and HDL-cholesterol were increased by silencing miR-144 in mice. In addition, studies in tissue-specific FXR deficient mice showed that hepatic but not intestinal FXR is essential for induction of miR-144 and FXR-dependent hypolipidemia. Interestingly, miR-144 was found to have sex-specific silencing effects [55]. Finally, miR-148a was found to control in vivo hepatic ABCA1 expression and circulating HDL-C levels, revealing a role for miR-148a as a key regulator of hepatic LDL-C clearance through direct modulation of LDLR expression, and showing the therapeutic potential of miR-148a inhibition to improve the elevated LDL-C/HDL-C ratio, a significant risk factor for cardiovascular disease [56].

Overall, these findings suggest that deregulated miRNAs can impact ABCA1 and RCT gene networks. These observations have generated singular interest in identifying novel targets for epigenetic regulation that may lead to novel strategies to raise functional HDL, promote RCT and help prevent atherosclerosis and CHD, which remains an essential challenge.

### 3.4. ABCA1, LDL-C and Triglyceride (TG) Serum Levels

While the effect of ABCA1 on HDL-C plasma levels is clear, the effect of ABCA1 loss of function on other lipid traits is less evident. Several authors report that TD patients have significantly elevated plasma TG levels and reduced LDL-C concentrations than normal controls, although plasma TG levels vary in these patients [57,58,59,60]. Clee et al. reported overall higher TG levels in subjects heterozygous for *ABCA1* mutations than in controls, although TG levels were variable and not elevated in all mutation carriers [61]. Using an extreme phenotype approach, Frikke-Schmidt et al. described nine patients heterozygous for *ABCA1* mutations with very low HDL-C levels, six of which had elevated TG levels (>2.2 mmol/L) [12]. In contrast, heterozygous carriers of *ABCA1* mutations have no significant change in LDL-C levels [62,63,64].

Most GWAS have not reported *ABCA1* as a locus associated with TG and LDL-C levels [65,66,67,68]. However, recent multiethnic GWAS, including hundreds of thousands of cases and controls, have identified different *ABCA1* variants associated with TG (rs2575876, rs1799777, rs1883025 and rs1800978) and LDL-C levels (rs7873387, rs2575876, rs2740488, rs11789603, rs2066714) with genome-wide significance, although with small effect sizes [69,70,71,72]. In one of these studies, associations with TG levels were observed in current drinkers and/or regular drinkers [69]. In addition, several candidate gene studies also reported *ABCA1* SNPs associated with LDL-C and TG levels, with inconsistent results. While various studies failed to find associations of *ABCA1* gene variation with TG and/or LDL-C levels [40,73,74,75], *ABCA1* polymorphisms were associated with TG levels but not with LDL-C levels in Brazilians [76], Chinese [77], Turkish [78], Iranians [79] and Mexican school-aged children [80]. The functional *ABCA1*/R230C variant was associated with lower triglyceride levels only in Pimas and Mayans, but not in Mexican mestizos [41]. Moreover, *ABCA1* gene variants have been associated with LDL-C but not TG levels in a cohort of Greek nurses [81], and in male individuals with hypercholesterolemia [82]. In a large multiethnic cohort studying over 150 common variants, *ABCA1* was associated with both TG and LDL-C levels [83].

Postprandial hypertriglyceridemia is an important factor in developing atherosclerotic plaque and is closely related to the occurrence of cardiovascular events [84,85]. Although TG levels are usually estimated in a fasting state, several epidemiological studies have demonstrated that non-fasting hyperlipidemia is more harmful [86,87,88]. The high interindividual variability of TG levels observed in TD may be due to the inherent heterogeneity in individual triglyceride levels in different postprandial dietary lipid absorption states [89,90]. While several studies have documented single candidate SNPs associated with postprandial TG metabolism modulation [91,92], studies analyzing the effect of *ABCA1* gene variants on postprandial lipid metabolism are scarce. A recent study identified that most of the interindividual variability in the postprandial chylomicron TG response to dietary fat in healthy male adults could be explained by a combination of 42 SNPs in 23 genes, including *ABCA1* [91]. Moreover, Delgado-Lista et al. showed that major allele homozygotes for rs2575875 and rs4149272 had lower postprandial increases in TG and large-triglyceride rich lipoproteins, suggesting these variants may regulate the clearance of postprandial triglycerides [93].

It is evident that altered ABCA1 function and gene variation do not always affect TG levels. This may have to do with ethnicity, sex-specific effects and with interactions with other gene variants and environmental factors. In this regard, a small number of studies have reported interactions between *ABCA1* gene variants and dietary macronutrient proportions affecting plasma TG levels. In the Mexican population, premenopausal women carrying the *ABCA1*/R230C risk allele and consuming higher carbohydrate/lower fat diets showed an unfavorable metabolic pattern including higher TG levels, with a statistically significant interaction [43]. An independent study in the Inuit population also reported an interaction between the *ABCA1*/R219K variant with saturated fat intake affecting plasma TG levels [94]. These facts indicate that gene-diet interactions may help better predict inter-individual variations in plasma lipid levels and may provide information useful to design diet intervention studies.

### 3.5. ABCA1 Gene Variation and Coronary Heart Disease

Because low HDL-C levels are a well-established independent risk factor for CHD, genetic variants known to increase HDL-C levels would be expected to decrease CHD risk, and variants associated with lower HDL-C levels would increase CHD risk. However, high HDL-C levels are not always protective of CHD, and Mendelian randomization studies suggest that the inverse relationship between HDL-C levels and CHD risk is not causal [95,96]. Possible explanations are differences in the functionality of HDL-C particles, and pleiotropic effects of ABCA1 [97]. Interestingly the R230C variant was found to be associated with both lower HDL-C levels and lower risk of premature coronary artery disease in the Mexican population [98]. While the possible effects of this and other variants on HDL-C functionality require further study, it is possible that the paradoxical effect of this variant could be due to a pleiotropic effect on platelet, endothelial and leukocyte-derived microparticle formation, all involved in atherosclerosis and CHD pathogenesis. This is the matter of ongoing research by our group.

## 4. ABCA1, Glucose Metabolism and Type 2 Diabetes

β-cell failure and insulin resistance in muscle and liver represent the core pathophysiologic defects in type 2 diabetes [99]. Although ABCA1 and cholesterol homeostasis are critical in β-cell function and play a role in insulin resistance, global loss of ABCA1 function is not enough to cause type 2 diabetes (T2D). Diabetes is not a characteristic feature of Tangier disease and was not a feature reported in global *Abca1*^−/−^ mice [100], although some consider diabetes as a complication of Tangier [101]. Moreover, while several patients suffering simultaneously from both diseases have been reported in the medical literature, particularly in the Japanese population [60,102,103,104], there are no reports on whether the prevalence of T2D is higher in Tangier patients. Still, several lines of evidence including tissue-specific *Abca1* KO models, human gene variation and ABCA1 expression studies point to a strong role of cholesterol homeostasis and ABCA1 in β-cell organization, function, and survival. Additionally, studies in muscle cell, hepatocyte and adipocyte-specific KO models, and some studies in humans, have shown ABCA1 also plays a role in peripheral insulin resistance. Altogether, this suggests that ABCA1 function is one of many factors which, acting in concert, contribute to the etiology of T2D.

### 4.1. ABCA1, Cholesterol and β-Cell Function

In addition to free fatty acid and triglyceride-mediated lipotoxicity, cholesterol toxicity is known to affect β-cell function and survival. β-cells are remarkably influenced by both intracellular cholesterol content and cholesterol distribution in the plasma membrane. Several transgenic and KO models have shown that increased cholesterol levels in β-cells reduce islet function, islet mass, and reduce insulin secretion by interfering with normal insulin secretory pathways [105,106,107,108,109]. In addition, cholesterol is important for maintaining the cholesterol-rich lipid rafts in the β-cell plasma membrane. By mediating the action of voltage-gated calcium channels and SNARE proteins, these lipid rafts mediate secretory stimuli and granule exocytosis/insulin secretion [110,111,112,113]. Being a cholesterol transporter affecting intracellular cholesterol concentrations and cholesterol membrane distribution, ABCA1 is thus expected to play a critical role in islet cholesterol homeostasis, β-cell function, insulin resistance and T2D.

Pancreatic β-cell specific *Abca1* KO mice (*Abca1*^-P/-P^) showed age-related and gene-dose-dependent accumulation of cholesterol in β-cells. In addition, these mice showed significantly decreased insulin secretion in response to an acute glucose challenge in vivo, along with progressive glucose tolerance impairment, which was not related to islet development or β-cell mass. The lack of ABCA1 in β-cells was later found to disrupt insulin granule exocytosis [109]. After loading *Abca1^−/−^* β-cells with cholesterol, Ca^2+^ influx in response to glucose stimulation decreased. These cells had a defective depolarization of the membrane and KCl-induced exocytosis. Interestingly, cholesterol depletion rescued the exocytotic defect in β-cells lacking ABCA1, supporting the notion that cholesterol accumulation plays an important role in the dysfunction of insulin secretion [109].

It is noteworthy that mice lacking *Abca1* specifically in β-cells have a more severe impairment in β-cell function compared with mice lacking *Abca1* globally. Because *Abca1*^-P/-P^ mice have higher levels of total plasma cholesterol than global *Abca1* KO mice, the degree of β-cell dysfunction caused by *Abca1* deficiency may be related to the level of plasma cholesterol to which the islets are exposed [105]. Thus, beneficial reductions in plasma lipids may limit the extent of β-cell damage [114].

### 4.2. ABCA1 and Insulin Sensitivity

Although global *Abca1^−/−^* KO mice did not show alterations in insulin sensitivity, multiple lines of evidence suggest that ABCA1 is involved in this trait. The interaction of HDL particles and ApoA-I results in the phosphorylation and activation of AMP-activated protein kinase (AMPK), a key metabolic enzyme that increases glucose uptake in murine endothelial cells, monocytes and skeletal muscle cells [115,116]. Similarly, in primary skeletal muscle cell cultures from T2D patients, HDL/ApoA-I bound to muscle cell surface receptors (including ABCA1), inducing intracellular Ca^2+^ mobilization, AMPK activation and glucose uptake. Antibody-mediated ABCA1 blockade inhibited HDL/ApoA-I glucose uptake and Ca^2+^ release in vitro, suggesting that HDL/ApoA-I modulates skeletal muscle glucose uptake in an ABCA1-dependent manner [117]. More recently, lipid-free ApoA-I was found to increase insulin-dependent and insulin-independent glucose uptake in primary human skeletal muscle cells, which were regulated by both ABCA1 and SR-B1, and this regulation seemed to be independent of ApoA-I acting as an acceptor of cellular cholesterol [118].

Moreover, observations in adipocyte-specific *Abca1*^-ad/-ad^ KO mice suggest a critical role for adipocyte intracellular cholesterol and ABCA1 in whole-body glucose homeostasis. These mice showed impaired glucose tolerance and lower muscle insulin sensitivity, along with significant changes in the adipose tissue expression of genes involved in cholesterol and glucose homeostasis, including *ldlr*, *abcg1*, *glut-4*, visfatin, adiponectin, and leptin. They also showed lower glucose-stimulated insulin secretion from β-cells ex vivo. Notably, reduced muscle-tissue insulin sensitivity and glucose tolerance were observed in *Abca1*-deficient mice fed a high fat, high cholesterol diet, suggesting that adipocyte ABCA1 is crucial for proper adipose tissue function in response to dietary fat and cholesterol [119]. Moreover, hepatocyte-specific *Abca1* KO mice (HSKO) produced a form of selective insulin resistance, suppressing lipogenesis but with normal glucose metabolism [120]. HSKO mice had reduced hepatic insulin-stimulated Akt phosphorylation, decreased SREBP-1c activation and reduced expression of lipogenic genes, but normal glucose and insulin tolerance.

### 4.3. ABCA1 Gene Variation and T2D

There are few studies analyzing β-cell function and insulin sensitivity in human heterozygotes for loss-of-function *ABCA1* mutations, most likely because these mutations are extremely scarce. In consistency with the mouse model, a small study (15 individuals with loss-of function *ABCA1* mutations vs 14 family controls) reported that heterozygosity for these mutations was associated with impaired insulin secretion, mild hyperglycemia and reduced first-phase insulin response to hyperglycemia. However, hyperglycemic clamp studies showed that mutation carriers had normal insulin secretion in response to an oral glucose challenge and had normal insulin sensitivity [64]. Notably, none of the *ABCA1* mutation carriers had diabetes, suggesting that heterozygosity alone confers a relatively mild susceptibility for diabetes. In contrast, a large study including 94 *ABCA1* heterozygotes from the Copenhagen City Heart and the Copenhagen General Population Studies did not find an association with increased T2D risk [121].

Similarly, associations of *ABCA1* polymorphisms with T2D are not always consistent. ABCA1 has not been reported to be significantly associated with T2D in genome-wide association studies [122,123,124]. However, candidate-gene studies have reported associations of *ABCA1* polymorphisms with T2D mostly in Asian and Latin American populations. Notably, several of these studies are small, including only hundreds of cases and controls. Daimon et al. (2005) were the first to report an association of *ABCA1* gene polymorphisms (a 34-SNP haplotype of the promoter region) with T2D in a small sample of the Japanese population [125]. A few years later, a functional variant (R230C), which decreases ABCA1 cholesterol efflux capability, was associated with early-onset T2D in two independent small cohorts of the Mexican population [126]. Interestingly, R230C was only marginally associated with T2D in Pimas [41], but significantly associated with T2D in Mayan individuals [127], and was not found to be associated with T2D in a case-control study of the Colombian population [128]. Moreover, the missense rs2230806 (R219K), frequently associated with higher HDL-C levels, was found to be associated with decreased T2D risk in a recent meta-analysis including Korean, Chinese and Indian individuals [129]. Several small studies have sought to associate rs1800997, a 5′UTR variant known as the C69T polymorphism, with T2D, with inconsistent results. The minor *ABCA1*/C69T allele was associated with decreased T2D risk in Turkish [130], Saudi [131], and Chinese Han [132] individuals, while the intronic rs4149313 variant was associated with increased T2D risk in a study including 8842 Koreans [133].

In addition to small sample sizes, which may limit statistical power, other factors could explain inconsistencies in studies seeking associations of *ABCA1* gene variation with T2D. According to observations in global and β-cell specific *Abca1* KO models, differences in serum lipid levels may be a determinant factor. Dyslipidemia is highly prevalent in the Mexicans [134], which is consistent with the association of the *ABCA1*/R230C variant with T2D in this population. Likewise, the association of this variant with lower total cholesterol and TG levels found in Pimas could be a factor explaining why it was only marginally associated with T2D in this group [41]. In addition, *Abca1* adipocyte and hepatocyte-specific KO models have shown that a high fat high cholesterol diet may influence the effect of ABCA1 impairment on certain traits [119,120]. In this regard, dietary macronutrient proportions have been found to modulate the effect of the *ABCA1*/R230C not only on lipid levels, but on other metabolic parameters such as homeostasis assessment model for insulin resistance (HOMA-IR), serum adiponectin levels and visceral to subcutaneous abdominal fat ratio [43]. In this study, lower proportions of dietary carbohydrate and higher proportions of dietary fat were associated with a more favorable metabolic profile in premenopausal women bearing the R230C variant. Because these gene-diet interactions were observed only in premenopausal women, gender effects on the associations with T2D are also likely.

## 5. ABCA1 and Liver Disease

The ABCA1 transporter is ubiquitous, is expressed in a wide variety of tissues and contributes importantly to the plasma HDL-C pool. Hepatic ABCA1 expression promotes cellular free cholesterol flow and improves RCT, transferring excess cholesterol from peripheral tissues to HDL and finally to the liver for the synthesis and excretion of bile acids [135,136].

Although *Abca1* gene inactivation in mice may increase lipid storage in hepatocytes and leads to the accumulation of sterols in some tissues [137,138,139], rare or common *ABCA1* gene variation seems not to be associated with nonalcoholic fatty liver disease (NAFLD). However, increased lipid and liver cholesterol deposition are known to play a role in the progression of steatosis to nonalcoholic steatohepatitis (NASH) [140,141,142]. Likewise, in patients with morbid obesity, Vega-Badillo et al. reported that miR-33a/144 hepatic expression and their target ABCA1 are associated with NASH [143]. Additional research is needed to conclude the role of ABCA1 in liver disease including its association with NAFLD/NASH.

## 6. ABCA1 in Neurological Disease

Cholesterol homeostasis is essential for the central nervous system (CNS). Approximately 23% of total body cholesterol is found in the CNS. Brain cholesterol is mainly synthesized in situ, as essentially no cholesterol enters the brain from the peripheral circulation [144]. Moreover, CNS growth and differentiation requires cholesterol produced by de novo synthesis [144,145]. The capability of neurons to biosynthesize cholesterol decreases in adulthood and depends mainly on glial cells [146]. ABCA1 is expressed in neurons and astrocytes, where it promotes the efflux of phospholipids and unesterified cholesterol to glia-derived apolipoprotein E (apoE) [147]. ApoE is the main apolipoprotein found and synthesized in the brain and is found in the interstitial and cerebrospinal fluid in the form of lipid-rich ApoE particles. The density and size of these particles are similar to those of plasma HDL [148]. ABCA1 contributes to cholesterol homeostasis and participates in the pathophysiology of neurological diseases involving the accumulation of proteins in brain cells, such as traumatic brain injury, stroke sequelae, Parkinson’s disease, and Alzheimer’s disease (AD) [149,150,151,152,153,154,155,156,157].

AD is a neurodegenerative disorder clinically characterized by progressive memory loss, disorientation and cognitive decline [158]. At the histopathological level, characteristic amyloid plaques and neurofibrillary tangles are found in the brain tissue [159,160,161]. Amyloid plaques develop from the accumulation of amyloid β peptide (Aβ) [161]. ApoE plays a crucial role in the proteolytic degradation of soluble forms of Aβ, and this effect is dependent of apoE lipidation by ABCA1-mediated cholesterol and phospholipid transfer [162]. The ABCA1 protein participates in this process by regulating apoE levels and function in the CNS [163,164,165,166,167].

In murine models, ABCA1 deficiency (*Abca1^−/−^*) was found to reduce apoE protein levels in the brain, to decrease lipidation of astrocyte-secreted apoE and to favor rapid apoE degradation [167,168]. *Abca1* deficiency may also increase amyloid burden in certain AD mouse models. Specifically, in a transgenic AD mouse model (APP23), targeted *Abca1* disruption (APP23/*Abca1^−^^/−^*) increased amyloid deposition, increased the level of cerebral amyloid angiopathy, exacerbated cerebral amyloid angiopathy-related microhemorrhage, and caused a sharp decrease of soluble, but not of insoluble brain apoE levels [167]. Conversely, selective ABCA1 overexpression in AD mouse models led to increased CNS apoE lipidation and sharply decreased amyloid deposition [168], while ABCA1 upregulation by miRNA-33 inhibition was found to increase apoE lipidation and to decrease Aβ levels in the brain [169]. Notably, Fitz et al. reported that while *Abca1* deletion in transgenic APP mice caused cognitive deficit at a stage of early amyloid pathology, these characteristics were not observed in *Abca1^−^*^/*−*^/wildtype mice. However, intra-hippocampal infusion of scrambled A oligomers affected cognitive performance of *Abca1* KO mice, which also showed altered neurite architecture in the hippocampus, suggesting that mice lacking ABCA1 have basal cognitive deficits that prevent them from coping with additional stressors [170].

Neuroinflammation and glucose metabolism are also important pathophysiological features in AD. Aβ deposits induce infiltration of immune cells such as T-helper 17 to the brain parenchyma and the secretion of proinflammatory cytokines such as interleukin 17A (IL-17A), which contribute to AD progression [171,172]. Interestingly, Yang et al. demonstrated that intracranial IL-17A overexpression increased ABCA1 protein levels in the hippocampus protein but not in cortex, decreased soluble Aβ levels in the hippocampus and cerebrospinal fluid, and improved glucose metabolism, suggesting that IL-17A may play a protective role in the pathogenesis of AD [173]. Moreover, hyperglycemic states are associated with greater severity of AD [174,175]. In this context, Lee et al. reported that in Zucker diabetic fatty rats (*fa^−^*/*fa^−^*) and in human neuroblastoma cells, exposure to high glucose levels increased Aβ deposition in the brain and decreased ABCA1 expression through JNK-reduced LXRα expression and binding to the *abca1* gene promoter [176].

Genetic studies support a role of ABCA1 in AD. Firstly, loss-of-function *ABCA1* mutations (N1800H) have been associated with low plasma apoE and increased AD risk in humans [177,178]. Moreover, although GWAS have consistently shown the crucial relevance of the *APOE4* variant in increasing AD risk across populations, *ABCA1* gene variation (rs3905000, rs27772082, rs2740488) has also been found to contribute to AD susceptibility in some GWAS [179,180,181,182,183,184,185]. Candidate gene studies analyzing the R219K polymorphism (rs2230806) and AD risk have reported conflicting results. This variant was associated with an increased risk of AD in Caucasian [186,187,188,189] and Chinese [51] populations, found to be a protective variant to AD in Chinese-Han and Hungarian individuals [190,191], and found not to be associated with AD risk in the German population [192]. However, two meta-analyses failed to find significant associations between *ABCA1* polymorphisms and AD [193,194].

It has been suggested that upregulation of ABCA1 expression or function may be a therapeutic target for AD and other diseases where Aβ plays a pathophysiological role. Interestingly, ABCA1 mediates the effect of some drugs proposed for AD treatment, such as bexarotene [195] and the liver X receptor agonist GW-3965 [164]. In addition, cyclodextrin [196], ondansetron [197], prostaglandin A1 [198], the purinergic receptor antagonist P2X7 [199], and the CS-6253 peptide [200] increase *ABCA1* gene expression in brain cells, although not all of these drugs improved cognitive function in vivo. Furthermore, Sarlak Z et al. reported that aerobic exercise significantly increases *Abca1* mRNA expression and decreases soluble Aβ1-42 in the hippocampus of rats with and without AD diagnosis. Aerobic training also improved cognitive function (learning and memory) [201]. ABCA1 and ApoE are currently the matter of intensive research for AD treatment [202].

## 7. ABCA1 and Microparticles

Microparticle (MP) release is a means for cell communication and cell-cell interaction, in addition to direct interaction and release of signaling molecules. MPs are small vesicles released from activated and/or apoptotic cells with substantial heterogeneity in size (50–250 nm). MPs include intracellular components involved in cell signaling and have membrane proteins characteristic of the original parent cell. It has been stablished that MPs are both biomarkers and cell signaling effectors that contribute to maintain and/or initiate cell dysfunction [203]. In a wide variety of thrombotic disorders, platelet and endothelial-derived MP levels are increased, with an interesting association between MP levels and pathophysiology, activity or progression of the disease [204]. MPs have procoagulant activity in several diseases including myocardial infarction [205,206], and may play a role in mediating inflammation-induced vascular calcification [207].

ABCA1 has a main role in facilitating outward bending or bulging of the plasma membrane [208]. It is currently known that the C-terminal of ABCA1 separately regulates its cholesterol floppase activity and cholesterol efflux activity [209]. Membrane dynamics are a prerequisite for HDL biogenesis and may also be required to release MPs to the medium [210]. ABCA1 and ApoA-I contribute to MP formation, mediating the production of MPs containing cholesterol. The addition of ApoA-I to human monocyte-derived macrophages markedly increased MP release, while ABCA1 inhibition with probucol and methyl-β cyclodextrin-induced membrane cholesterol depletion markedly reduced MP release and nascent HDL formation. MPs do not contain ApoA-I, but contain the plasma membrane marker flotilin-2, and CD63, an exosome marker. ABCA1 promotes cholesterol efflux, reduces cellular cholesterol accumulation and regulates anti-inflammatory activities in an ApoAI or annexin A1 (ANXA1)-dependent manner. ABCA1 anti-inflammatory activity seems to occur by mediating the efflux of ANXA1, which plays a critical role in anti-inflammatory effects, cholesterol transport, exosome and microparticle secretion and apoptotic cell clearance [211].

Although many studies have shown the importance of *ABCA1* gene variation in serum HDL-C levels, very few studies have reported the effect of gene variants on MP formation and their possible clinical consequences. It is known that ABCA1 participates in infectious and/or thrombotic disorders involving vesiculation [212], and in vitro studies and animal models indicate that ABCA1 also plays an important role in MP formation [21,208]. In Hamster kidney cells and mouse macrophages, ABCA1 was found not only to promote cholesterol efflux towards ApoA-I forming nascent HDL, but it also promoted the formation of ApoA-I-free MPs. This study also demonstrated that the *ABCA1* A937V mutation altered the formation of HDLs and concurrently reduced the release of MPs [208]. Moreover, in an experimental mouse model of cerebral malaria, Combes et al. evaluated the pathogenic implications of MP using *Abca1* deficient mice. Upon infection by *Plasmodium berghei ANKA*, these mice showed complete resistance to cerebral malaria, and MPs purified from infected animals were able to reduce normal plasma clotting time and to significantly enhance tumor necrosis factor release from naive macrophages [213,214]. *ABCA1* promoter variants associated with increased atherosclerotic burden [73] were found to be associated with decreased MP levels and were more prevalent in patients with uncomplicated malaria, suggesting that these polymorphisms have a protective effect against severe malaria in humans [215].

Calcium-dependent cytoskeleton proteolysis causes an eventual transient phospholipid density imbalance between the two plasma membrane leaflets driven by swift phosphatidylserine (PS) egress and lower reverse transport of phosphatidylcholine and sphingomyelin. This imbalance causes local instability of the plasma membrane and MP release upon raft clustering. The calcium-dependent channel TMEM16F plays a crucial role in calcium-induced phospholipid scrambling in the release of MPs exposing PS. TMEM16F mutations cause Scott Syndrome, a rare bleeding disorder characterized by defective platelet PS membrane exposure and MP shedding [216,217,218]. Because ABCA1 is known to have a role in exofacial PS translocation, Albrecht et al. analyzed the role of this protein in the pathophysiology of a Scott Syndrome patient who carried an *ABCA1* mutation (R1925Q). In vitro expression studies revealed that the 1925Q variant showed impaired trafficking to the plasma membrane, while wild-type ABCA1 overexpression in Scott Syndrome lymphocytes complemented the calcium-dependent PS exposure at the cell surface. Thus, this *ABCA1* mutation contributed to the defective PS translocation phenotype [219].

*Abca1*-deficient mice show alterations in PS exposure and significant reductions in circulating levels of MPs [212,220]. Moreover, silencing of *ABCA1* in human umbilical cord endothelial cell (HUVEC) cultures significantly reduced the release of MPs when subjected to frictional forces [221]. In this study, atheroprone shear stress conditions stimulated the formation and release of endothelial-derived MPs and hemodynamic forces were identified as an important determinant of MP plasma levels in healthy subjects. Sustained exposure to atheroprone low shear stress conditions increased both endothelial apoptosis and the release of MPs in the medium, when compared with physiological high shear stress conditions. Moreover, downregulation of ABCA1 expression by endogenously released nitric oxide (NO) contributed to limit the release of endothelial-derived MPs in HUVECs exposed to high shear stress [221].

## 8. ABCA1 in Infectious Diseases

The ABCA1 protein plays an important role in the development of some infectious diseases because of its role in cholesterol metabolism [222]. ABCA1 expression can be altered by some viruses, parasites and bacteria including components of the intestinal microbiota, [1,155,212,223,224,225,226], and some authors have proposed ABCA1 as a possible therapeutic target for these infections [212,227,228]. The entry and exit sites of some viral agents such as the human immunodeficiency virus, hepatitis C virus and cytomegalovirus occur in cholesterol, phospholipid and transporter enriched microdomains called lipid rafts [227,229,230,231]. The interaction of ABCA1 with these viruses alters lipid metabolism and intracellular signaling pathways [225,231,232].

### 8.1. Human Immunodeficiency Virus (HIV)

HIV is a retrovirus that infects and depletes CD4 T lymphocytes, causing slowly progressive immunodeficiency [225]. Despite antiretroviral therapy, people infected with HIV continue to develop comorbidities such as dyslipidemia, atherosclerosis and diabetes [228].

**Figure 2:**
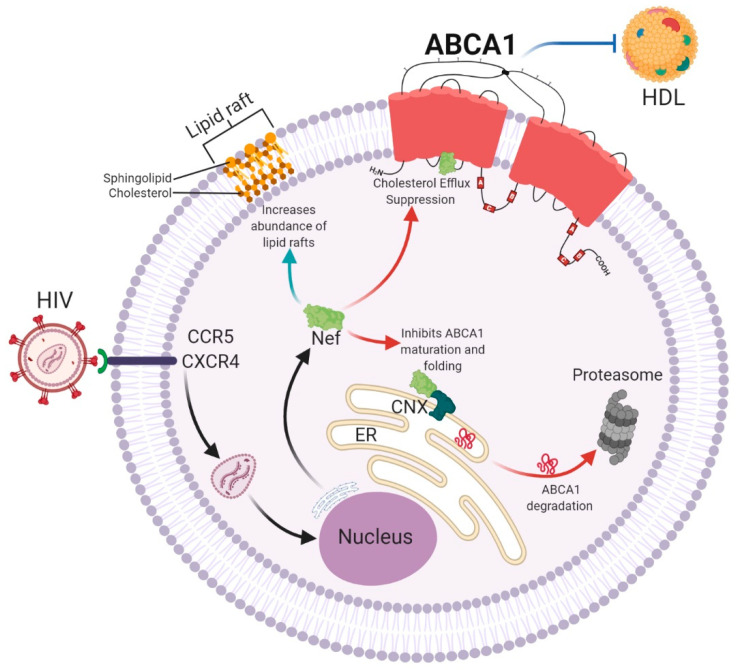
Effects of the viral negative factor (Nef) protein in human immunodeficiency virus (HIV)-infected cells. HIV enters cells by binding to the chemokine receptor 5 (CCR5) and chemokine receptor type 4 (CXCR4) and uses the host cell machinery to synthesize viral proteins such as Nef. Nef increases cholesterol biosynthesis and induces lipid raft formation, required to produce new virions. Nef also inhibits cholesterol efflux by suppressing ABCA1 activity, inducing structural and functional modifications of high-density lipoproteins (HDL). In addition, Nef blocks the interaction of the endoplasmic reticulum (ER) chaperon calnexin (CNX) with ABCA1, altering its folding and maturation. Nonfunctional and misfolded ABCA1 is retained in the ER and degraded in the proteasome, resulting in further accumulation of intracellular cholesterol, creating a favorable microenvironment for viral replication and release.

The role of the viral negative factor (Nef) protein and its association with cardiometabolic comorbidities has become of great interest in recent years (Figure 2). Nef is a multifunctional viral protein that alters the expression of different macromolecules on the surface of the host cell [233]. Nef decreases *ABCA1* gene expression, increases ABCA1 protein degradation in lysosomes and proteasomes by displacing it from the lipid rafts, and alters its maturation and folding in the endoplasmic reticulum by blocking its interaction with calnexin [234,235,236,237,238]. These events induce the accumulation of intracellular cholesterol in the host cell and increase the number of nonfunctional lipid rafts allowing virus survival and increasing virion production [228,239,240,241,242]. In addition, recent studies have shown that Nef can be released from infected cells through extracellular vesicles altering cholesterol metabolism in uninfected recipient cells [237,243,244,245].

It is well known that HIV patients develop dyslipidemia, and their HDL-C plasma concentrations can be as low as those of TD patients [60,246,247]. The Nef protein causes dyslipidemia, as it affects cholesterol efflux by reducing the expression of ABCA1 in in vitro and in vivo models [237,248,249,250]. Similarly, the accumulation of cholesterol in pancreatic β-cells alters their function, decreasing insulin release, predisposing HIV patients to diabetes [105,109,251]. In this context, some studies have shown that antiretroviral therapy not only reduces the viral load [249] but also increases ABCA1 expression, restoring cholesterol efflux and increasing HDL-C plasma concentrations [249,252]. A recent prospective study reported that new antiretroviral therapies mitigate the cardiometabolic effects of HIV, at least in the short term [246]. However, not all studies report cardiometabolic improvement [253,254]. Moreover, long-term antiretroviral therapy is associated with dyslipidemia, although it does not occur in all patients [255]. A study assessing the impact of 192 SNPs in HIV patients receiving antiretroviral therapy identified that the *ABCA1* rs4149313 was associated with decreased TG and increased HDL-C circulating levels [256], while an independent study reported that *ABCA1* rs2066714 was associated with a greater risk of dyslipidemia in patients under antiretroviral treatment [257]. In addition, because of the role of ABCA1 in viral replication, ongoing studies are also investigating whether functional ABCA1 gene variants affect HIV progression or severity.

### 8.2. Hepatitis C Virus (HCV)

HCV belongs to the *Flaviviridae* family, has marked tropism for liver parenchymal cells, and chronic HCV infection leads to liver cirrhosis and hepatocellular carcinoma [227,232,257]. ABCA1 was found to have low expression levels in hepatocellular carcinoma samples from patients with a history of HCV infection [257]. Consistently, HCV infection was found to increase miR-27a expression in vitro. This miRNA binds to the 3′UTR sequence of *ABCA1* mRNA decreasing ABCA1 protein levels [258]. These events increase cellular cholesterol content and promote virus replication [232,259]. Furthermore, pharmacologically-induced *ABCA1* gene expression caused lipid raft reorganization in human hepatocytes, inhibiting HCV infection [227].

### 8.3. Human Cytomegalovirus (HCMV) and Other Viruses

HCMV is an opportunistic pathogen associated with an increased risk of atherothrombosis. In vitro models demonstrated that HCMV infection and the viral US28 protein decrease ABCA1 expression in the host cell, altering the distribution of lipid-rich microdomains in the plasma membrane [229,260]. Finally, although cholesterol metabolism has been found to be altered in other viral infections such as dengue [261,262] and chikungunya [263], the direct participation of ABCA1 in these diseases has not been demonstrated [261,263,264].

### 8.4. Malaria

Malaria is a parasitic disease caused by *Plasmodium* infection, transmitted to humans through the bite of infected female *Anopheles* mosquitoes [265]. Cerebral malaria is a severe complication, occurring in approximately 1% of those infected, and is the main cause of death [266]. The role of ABCA1 in microvesicle formation seems to be relevant in the pathogenesis of cerebral malaria. *Abca1* KO mice showed lower plasma concentrations of microvesicles, and when infected with *Plasmodium berghei ANKA*, these mice show complete resistance to cerebral malaria. In addition, plasma tumor necrosis factor alpha concentrations were reduced, decreasing the proinflammatory and prothrombotic state [212]. Furthermore, *ABCA1* promoter variants were associated with increased microvesicle production and a higher risk of developing severe malaria in humans, suggesting that *ABCA1* genetic variation may confer susceptibility to the development of malaria and its complications [215]. Because miR-27a was found to inhibit *ABCA1* expression in vitro, to abolish microvesicle production and inhibit apoptotic mechanisms, this miRNA has been proposed as protective against cerebral malaria [267,268]. Interestingly, in a murine model of malaria infection during pregnancy, *Abca1* expression was increased in the endothelial cells of the yolk sac. This event may be the result of a compensatory mechanism to maintain cholesterol homeostasis and favor the development and survival of the fetus [269]. Thus, ABCA1 may have a dual role, sometimes favoring infection and sometimes conferring protection.

Further research is required to fully elucidate how ABCA1 and cholesterol homeostasis are involved in infections, and to establish whether ABCA1 can in fact be a therapeutic target.

## 9. ABCA1, Age-Related Macular Disease and Glaucoma

Age-related macular degeneration (AMD) is a leading cause of visual impairment and severe vision loss in individuals above 50 years of age. AMD is a multifactorial and complex disorder, where immunological factors, inflammation, lipid and cholesterol metabolism, angiogenesis and extracellular matrix are involved in the disease pathogenesis [270]. Early disease is characterized by the presence of cholesterol-rich extracellular deposits similar to atherosclerotic plaques underneath the retinal pigment epithelium (RPE) or in the subretinal space, called drusen or drusenoid deposits [271,272,273,274,275,276], which may lead to atrophic neurodegeneration or pathologic angiogenesis. Drusen contain polar lipids such as free cholesterol and phosphatidylcholine, as well as neutral lipids such as cholesteryl esters and apolipoproteins [277]; while drusenoid deposits seem to contain only free cholesterol and apolipoproteins [278].

Several lines of evidence suggest that cholesterol metabolism and ABCA1 are involved in AMD pathogenesis. Systemic disturbances in cholesterol metabolism causing altered lipoprotein subtype levels have been associated with AMD [279]. Moreover, while retinal abnormalities have not been reported in Tangier disease, GWAS and candidate gene studies have shown that *ABCA1* gene variation contributes to AMD susceptibility, although to a lesser degree than the complement factor H (*CFH*) Y402H and age-related macular susceptibility-2 (*ARMS2*) A69S polymorphisms, which are well established risk factors for AMD [280,281,282,283,284,285]. In addition, human RPE cells express *ABCA1* and other genes involved in lipid metabolism such as *SRBI*, and glyburide-mediated inhibition of ABCA1 and SRBI activity was found to abolish HDL-stimulated basal efflux of photoreceptor-derived lipids in cultured human RPE cells, supporting a role of RCT regulation in the pathogenesis of AMD [286]. Finally, murine KO models also support the role of ABCA1 and cholesterol metabolism in AMD pathogenesis. Targeted deletion of macrophage *Abca1* and *Abcg1* in mice led to age-associated extracellular cholesterol-rich deposits underneath the neurosensory retina similar to the drusenoid deposits observed in early stages of human AMD, and the mice developed impaired dark adaptation and rod photoreceptor dysfunction [287,288].

Glaucoma is the world’s leading cause of irreversible blindness [289]. It is a degenerative optic neuropathy characterized by the progressive degeneration of retinal ganglion cells (RGC) and the retinal nerve fiber layer (RNFL), leading to visual impairment and eventually to blindness. Elevated intraocular pressure (IOP) is a major risk factor for most types of glaucoma. Primary open-angle glaucoma (POAG) is characterized by increased resistance to aqueous fluid outflow through the trabecular meshwork and is the most common form of glaucoma worldwide. Primary angle-closure glaucoma is caused by blocked access to the outflow tracks; and secondary exfoliation glaucoma is a sequela of exfoliation syndrome characterized by accumulation of a characteristic fibrillar material on the ocular lens and trabecular meshwork [290]. Mendelian forms of glaucoma are caused by mutations in *MYOC*, *OPTN* and *TBK1* genes [291]. However most cases of glaucoma are multifactorial, and various biological processes including lipid metabolism, cytokine signaling, membrane biology, extracellular matrix, fucose and mannose metabolism, cell and ocular development are involved in the pathophysiology of the disease.

Several lines of evidence including GWAS, animal models and in vitro studies suggest ABCA1 plays an important role in the pathophysiology of glaucoma, mainly POAG. Although glaucoma is not a characteristic of Tangier disease, GWAS for IOP and POAG have identified common variants in or near *ABCA1* (rs2472493 and rs2487032) among more than 50 loci in Asian and European Caucasian populations [292,293,294,295]. However, while ABCA1 and other genes involved in lipid metabolism were found to be associated with IOP and POAG, a Mendelian randomization study did not find any evidence for a causal association between plasma lipid levels and POAG risk [296].

*ABCA1* is highly expressed in retinal ganglion cells and its expression is significantly higher in individuals with glaucoma and upregulated in high-IOP glaucoma murine models. This suggests that ABCA1 is involved in the normal biological functions and cell death of ganglion cells. A recent study reported evidence of a novel role for ABCA1 in IOP modulation via the regulation of the Cav1/eNOS/NO signaling, which is likely to be an important mechanism of pathogenesis in patients with POAG. Based on their findings, the authors suggest that enhancing the ABCA1 signaling pathway could be of therapeutic value in the treatment of glaucoma and ocular hypertension [297].

## 10. ABCA1 in Cancer

Cellular cholesterol homeostasis is highly regulated to maintain cell membrane integrity and to promote membrane-anchored signaling pathways, and this homeostasis is altered during cancer cell proliferation. Epidemiologic studies have associated high serum total cholesterol concentrations with decreased risk of cancer [298,299]. Furthermore, tumor cells have been found to show high levels of cholesterol, suggesting that cholesterol metabolism is increased in proliferating cancer tissues [300,301]. ABCA1-mediated cholesterol efflux is one of the major regulation pathways of cholesterol. Moreover, as cancer is a highly cooperative process of oncogenic mutations that causes multiple metabolic changes including changes in gene expression patterns, *ABCA1* was identified as one of the cooperation response genes, nonmutant genes synergistically downregulated by multiple cancer gene mutations in the processes of malignant cell transformation [302]. Thus, numerous studies have investigated the role of ABCA1 in cancer development.

There may be a dual role of ABCA1 in cancer, as several studies suggest that ABCA1 function has anticancer properties, although there is also epidemiological and experimental evidence suggesting it may be involved in progression of certain types of cancer. On one hand, diminished *ABCA1* expression in neoplastic breast and prostate tissue was associated with an increased rate of cancer cell proliferation [303,304]. Likewise, ABCA1 downregulation caused by *ABCA1* promoter hypermethylation, miR-183 degradation or loss of function mutations, led to elevated cholesterol levels in cancer cells, enhanced cell proliferation and inhibited apoptosis [305,306,307,308,309]. On the other hand, *ABCA1* has been classified as a member of a lipid metabolism gene expression signature (ColoLipidGene) related to poor prognosis in patients with colorectal cancer (CRC). This signature includes four overexpressed lipid metabolism-related genes [310]. ABCA1 has been proposed as a specific marker of triple-negative breast cancer (TNBC) since its expression was higher in TNBC tissues compared with noncancerous mammary tissues [311]. Additionally, high-level expression of ABCA1 in primary tumors of serous ovarian cancer was associated with reduced survival of the patients and enhanced tumor cell growth and migration [312].

**Figure 3:**
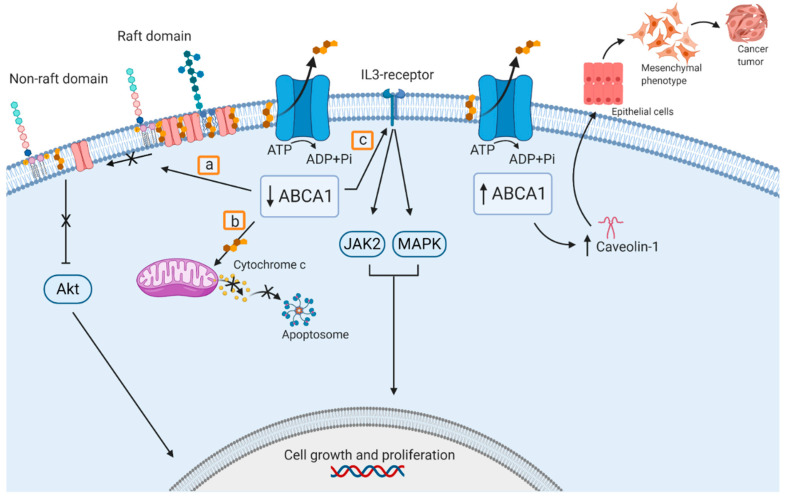
Proposed pathways of ABCA1 involvement in cancer. Downregulation of ABCA1 promotes cell growth and proliferation. a: ABCA1 downregulation avoids raft domain disruption and Akt pathway activation; b: ABCA1 downregulation causes cholesterol accumulation in the mitochondrial membrane inhibiting cytochrome c release and apoptosome formation; and c: ABCA1 downregulation activates the IL3-receptor, activating Janus kinase 2 (JAK2) and mitogen-activated protein kinase (MAPK) pathways. Upregulation of ABCA1 stabilizes caveolin-1 promoting epithelial-mesenchymal transition, and thus cell migration an invasion.

Several groups have provided insights into the molecular mechanisms of ABCA1 in cancer biology to help understand its pathophysiology and to identify potential therapeutic targets. Among the mechanisms proposed for ABCA1 anticancer activity are the following. (1) Deficient ABCA1-mediated cholesterol efflux increases intracellular and mitochondrial cholesterol levels, which decreases mitochondrial membrane fluidity and inhibits mitochondrial permeability transition. This avoids the release of cell death-promoting molecules such as cytochrome c and the apoptosis-inducing factor [308,313]. (2) ABCA1 activity has been linked to lipid raft disruption, by redistributing cholesterol and sphingomyelin from raft to nonraft domains. This results in reduced Akt signaling activation, which is sensitive to raft integrity. Akt upregulation has been associated with prostate cancer progression [314,315]. In other words, ABCA1 downregulation causes Akt upregulation, which in turn promotes cancer cell growth. (3) ABCA1 is known to suppress hematopoietic cell proliferation. Somatic *ABCA1* mutations found in chronic myelomonocytic leukemia patients were found to impair cholesterol efflux and increase cell proliferation by enhancing the cholesterol-dependent IL3-receptor β pathway, which activates and protein-tyrosine kinase Janus kinase 2 (JAK2) and mitogen-activated protein kinase (MAPK) signaling [309] (Figure 3).

In contrast, other studies proposed mechanisms associated with ABCA1 activity in favor of cell cancer proliferation. For example, in colorectal cancer cell lines, ABCA1 overexpression led to an epithelial-to-mesenchymal transition and stabilized caveolin-1, known to promote cell migration, invasion, and has been proposed to be involved in tumor cell metastasis [316]. In addition, downregulated ABCA1 expression was found to prevent melanoma and bladder tumor growth in a syngeneic murine melanoma tumor model with a myeloid-specific *Abca1* deletion. Lack of Abca1 inhibited tumor bed accumulation of myeloid derived suppressor cells, known to promote tumor angiogenesis, metastasis and immune evasion, resulting in tumor growth inhibition [317].

Summarizing, although there is no conclusive evidence that ABCA1 is involved in the carcinogenesis process, unlike other members of the ABC transporter family (reviewed in [318]), it seems to play an important role in proliferation and survival of cancer cells. Moreover, while most studies suggest that ABCA1 activity is protective of cancer progression, there is also evidence of ABCA1 facilitating cell proliferation and tumor growth. Thus, the consequences of ABCA1 down or upregulation should be thoroughly investigated in different types and stages of cancer. Since intracellular cholesterol accumulation plays a key role in cancer progression, ABCA1 has been proposed as a potential therapeutic target; nevertheless, this subject needs further investigation.

## 11. Concluding Remarks

By regulating cholesterol homeostasis and plasma membrane dynamics, ABCA1 is involved in many physiological and pathological processes. ABCA1 protects cells from cholesterol toxicity by promoting cholesterol efflux. In addition, by regulating plasma membrane dynamics, ABCA1 plays a role in cell signaling and microparticle formation. Through these mechanisms, ABCA1 is involved in the pathogenesis of a broad array of diseases including dyslipidemia, atherosclerosis, coronary heart disease, type 2 diabetes, thrombosis, neurological disorders, age-related macular degeneration, glaucoma, viral infection, and cancer progression. However, most of these diseases have a complex etiology, where ABCA1 function is one of several factors playing a role in the pathophysiological process. Finally, although ABCA1 has been proposed as a therapeutic target for Alzheimer’s disease, age-related macular degeneration, viral infections and other diseases, because the level of pleiotropy of this protein is high, tissue specific ABCA1 targeting may be important to achieve the desired therapeutic effect. A great deal of research is needed to further understand its physiological and pathological role, and the possibilities of targeting ABCA1 for therapy.
